# Alteration of the gut microbiome in mycophenolate-induced enteropathy: impacts on the profile of short-chain fatty acids in a mouse model

**DOI:** 10.1186/s40360-021-00536-4

**Published:** 2021-10-28

**Authors:** Manon Jardou, Quentin Provost, Clarisse Brossier, Émilie Pinault, François-Ludovic Sauvage, Roland Lawson

**Affiliations:** 1 Univ. Limoges, Inserm U1248, IPPRITT, F-87000 Limoges, France; 2 Faculté de Pharmacie, Université de Limoges, 2 rue du Dr Marcland, 87025 Limoges, France

**Keywords:** Mycophenolate-induced enteropathy, Gut microbiome, Short-chain fatty acids, Acetate, Propionate, Butyrate

## Abstract

**Background:**

Mycophenolic acid (MPA) is the most widely used immunosuppressive drug in transplantation and for autoimmune diseases. Unfortunately, more than 30% of patients experience a typical gastrointestinal adverse effect also referred to as mycophenolate-induced enteropathy. Due to its antibacterial, antifungal, and antiviral properties, MPA exposure is associated with intestinal dysbiosis characterized by a decrease in density and diversity of the microbiome regarding the main bacterial phyla (Firmicutes and Bacteroidetes). These bacterial phyla are known for their metabolic role in maintaining the homeostasis of the digestive tract, particularly through the production of short-chain fatty acids (SCFA) that could contribute to the pathophysiology of mycophenolate-induced enteropathy. Our study aimed at deciphering short-chain fatty acids (SCFA) profile alterations associated with gastrointestinal toxicity of MPA at the digestive and systemic levels in a mouse model.

**Methods:**

Ten-week old C57BL/6 (SOPF) mice were randomly assigned in 2 groups of 9 subjects: control, and mycophenolate mofetil (MMF, 900 mg/kg/day). All mice were daily treated by oral gavage for 7 days. Individual faecal pellets were collected at days 0, 4 and 8 as well as plasma at day 8 for SCFA profiling. Additionally, after the sacrifice on day 8, the caecum was weighted, and colon length was measured. The proximal colon was cut for histological analysis.

**Results:**

MMF treatment induced around 10% weight loss at the end of the protocol associated with a significant decrease in caecum weight and a slight reduction in colon length. Histological analysis showed significant architectural changes in colon epithelium. Moreover, we observed an overall decrease in SCFA concentrations in faecal samples, especially regarding acetate (at day 8, control 1040.6 ± 278.161 μM versus MMF 384.7 ± 80.5 μM, *p* < 0.01) and propionate (at day 8, control 185.94 ± 51.96 μM versus MMF 44.07 ± 14.66 μM, *p* < 0.001), and in plasma samples for butyrate (at day 8, control 0.91 ± 0.1 μM versus MMF 0.46 ± 0.1 μM, *p <* 0.01).

**Conclusions:**

These results are consistent with functional impairment of the gut microbiome linked with digestive or systemic defects during MMF treatment.

**Supplementary Information:**

The online version contains supplementary material available at 10.1186/s40360-021-00536-4.

## Background

Mycophenolic acid (MPA) is one of the most widely used immunosuppressants in transplantation and for the treatment of some autoimmune diseases [1–3]. MPA, used as a prodrug (mycophenolate mofetil or MMF) or a salt (mycophenolate sodium), is a potent, selective, non-competitive and reversible inhibitor of inosine-5′-monophosphate dehydrogenase 2 (IMPDH2). IMPDH2 plays a key role in the de novo purine nucleotide synthesis, on which lymphocytes rely for DNA biosynthesis. Therefore, MPA exerts preferentially a cytostatic effect in T and B lymphocytes, which do not possess the recovery pathway for DNA biosynthesis through hypoxanthine-guanine phosphoribosyltransferase (HPRT) [4]. After oral absorption, MPA enters the systemic circulation where it directly acts on circulating lymphocytes. MPA can also reach lymphoid tissues (e.g., bone marrow, thymus, lymph nodes and spleen) for modulating in situ lymphocyte differentiation and activation [5, 6].

MPA treatment is unfortunately associated with adverse effects, among which gastrointestinal disorders are of major concern [7]. More than 30% of patients experience gastrointestinal adverse effects, ranging from nausea, vomiting, abdominal pain or diarrhoea, to erosions of the gastrointestinal tract with life-threatening bleeding ulcerations [8, 9]. Histologically, the lesions are characterized by architectural disorganization of the digestive epithelium mainly in the colon. Oedema and/or inflammation of the *lamina propria* are also observed, mimicking the inflammatory lesions of graft-versus-host disease in bone marrow transplantation [7, 10–12].

Recent investigations have pointed to the alteration of the gut microbiome, also referred to as intestinal dysbiosis, by immunosuppressive agents. Moreover, intestinal dysbiosis is responsible for abnormal gut microbiome β-glucuronidase activity and increased gastrointestinal exposure to MPA in mycophenolate-induced enteropathy [13–16]. MPA was first isolated as a fermentation product of *Penicillium stoloniferum* and was initially known for its antibacterial, antifungal, and antiviral properties [4]. Therefore, gastrointestinal tract exposure to high concentrations of MPA is expected to be associated with intestinal dysbiosis characterized by a decrease in density and diversity of the gut microbiome regarding the main bacterial phyla (e.g., Firmicutes and Bacteroidetes) which has been recently validated with a mouse model [15, 16]. These bacterial phyla are known for their metabolic role in maintaining the homeostasis of the digestive tract, particularly through the production of short-chain fatty acids (SCFA) from the fermentation of indigestible carbohydrates [17–20]. The three main SCFA (acetate, propionate and butyrate) can passively penetrate and pass through intestinal cells. SCFA can also be actively incorporated through sodium-coupled monocarboxylate transporter 1 (SMCT1) or monocarboxylate transporter 1 (MCT1) located on the apical side of intestinal epithelial cells and transported into the bloodstream through monocarboxylate transporter 4 and 5 (MCT4 and MCT5) found on the basolateral side [21–23]. SCFA are pharmacologically active and can selectively regulate three different G-protein coupled receptors (GPCR) on intestinal cells, namely, GPR41 (free fatty acid receptor 3 or FFAR3), GPR43 (free fatty acid receptor 2 or FFAR2) and GPR109A (hydroxycarboxylic acid receptor 2 or HCAR2) [24–26]. These receptors are also expressed on various other cell types in organs and tissues such as the liver, muscles, neurons and immune cells (T and B cells, macrophages) [27, 28]. SCFA function as “co-hormones” as they are produced by the gut microbiome, which is assimilated to a metabolic organ. Intracellular SCFA can exhibit a strong histone deacetylase (HDAC) inhibitory effect, with butyrate being the most potent inhibitor [29–32]. Histone hyperacetylation is associated with increased accessibility of promoter regions and genes modulation [33]. For example, butyrate through its HDAC inhibitory activity, can imprint an antimicrobial program in macrophages and thus, eliminate invasive pathogens and regulate the inflammatory response [34]. Butyrate can also be a source of energy for the colonocytes and can improve epithelial barrier functions through up-regulation of tight junctions proteins such as Claudin-1 and ZO-1 [35, 36]. SCFA may also have beneficial effects on gut homeostasis by enhancing mucosal barrier integrity through Mucin-2 expression [37]. Hence, the functional alterations of the SCFA profile can be involved in the pathophysiology of the mycophenolate-induced enteropathy. Our study aimed at analyzing the alterations of the SCFA profile at the local and systemic levels and their associations with the gastrointestinal toxicity of mycophenolic acid in a mouse model.

## Methods

### Experimental animals

In this study, our experimental model is inspired by the pioneering work published by Flannigan et al., 2018 [16] and Taylor et al., 2019 [15], which demonstrated an impairment of the gut microbial community associated with colon tissue inflammation under MPA treatment. For this purpose, female C57BL/6 J adult (10 weeks old) Specific and Opportunistic Pathogen Free mice purchased from Janvier Labs (Saint-Berthevin, France) were used for in vivo experiments and were housed in a pathogen-free animal facility, with 12 h/12 h of light/dark and unrestricted access to food and water. The Regional Ethics Committee for Animal Experimentation (CEEA-033, Région Limousin) and the French Ministry of Higher Education, Research and Innovation, under Project Authorization for the use of Animals for Scientific Purposes 23,785-2,020,012,416,174,896 v3, approved animal care and experimental procedures. These experimental procedures were performed in accordance with the guidelines for animal experimentation of the European Communities Council Directive (EU/63/2010) and reported according to the ARRIVE guidelines [38]. Eighteen mice were randomly assigned into two groups: control (*n* = 9) and MMF (*n =* 9) and treated for 7 days. Mice were daily monitored for body weight. MMF treatment (900 mg/kg/day of a clinical formulation of MMF (Cellcept®) dissolved in saline solution) or saline solution (control group) were given by oral gavage with a 20 Gauge dosing cannula. For the sacrifice, the mice were anaesthetized with intraperitoneal injection of pentobarbital (100 mg/kg, CEVA Santé Animale, France).

### Sample collection and storage

#### Faeces

Individual faecal pellets were collected using metabolic cages at day 0, day 4 and day 8 and were placed in a sterile collection tube and stored at − 80 °C for SCFA analysis.

#### Plasmas

Peripheral blood was obtained using a 22 Gauge heparinised syringe from anaesthetised mice on day 8 by intracardiac puncture and plasma was prepared by centrifugation at 10,000 g for 10 min at 4 °C and stored at − 80 °C for SCFA analysis.

#### Caecum/colon block

The block caecum/colon was harvested after sacrifice. The caecum was weighted and colon length measured. The proximal colon (around 1 cm) was cut and placed in a 4% formaldehyde solution for histological analysis.

### SCFA quantification

#### Samples preparations

To quantify the SCFA in faecal samples, 25 μL of 50% aqueous acetonitrile solution was added to 1 mg of faeces, vortex mixed for 10 min and then placed for 10 min in an ultrasonic bath. The samples were centrifuged at 10,000 *g* at 10 °C for 10 min. The supernatants were collected. This procedure was repeated twice for each faecal sample and the supernatants were pooled. For plasma SCFA analysis, the samples were directly diluted 1:5 with a saline solution. Calibration standards (acetate from 10 to 5000 μM, propionate from 1 to 500 μM, butyrate from 0.4 to 200 μM) were prepared either in 50% aqueous acetonitrile for faecal samples or in saline solution for plasma samples.

The SCFA derivatization step was based on a previously published method [39]. Briefly, 50 μL of each sample (pooled supernatants for faeces or diluted plasma) were mixed with 20 μL of 200 mM 3-NPH, 20 μL of 120 mM EDC-6% pyridine, both solubilised in 50% aqueous acetonitrile solution, and 20 μL of pooled internal standard solution (D4-acetic acid 10 mM, D2-propionic acid 0.4 mM, D7-butyric acid 0.25 mM diluted in ultrapure water). The reaction was performed at 40 °C for 30 min in the dark and the samples were then diluted 1:20 (v/v) with 10% aqueous acetonitrile, and 1 μl or 5 μl thereof (for faeces or plasma derivation mixture respectively) were analysed by LC-MS/MS.

#### LC-MS/MS analysis

Briefly, chromatographic separation was performed using a Shimadzu Nexera 2 LC system (Shimadzu Corporation, Marne-la-Vallée, France) equipped with a thermostated column compartment and a thermostated microwell plate autosampler with a six-port micro-switching valve. LC separation was performed on an Accucore™ RP-MS column (100 × 2.1 mm, 2.6 μm solid core, Thermo Scientific) at a flow rate of 0.2 mL/min at 60 °C. The mobile phase was a gradient of 0.1% formic acid in water (solvent A) and 0.1% formic acid in methanol (solvent B) programmed as follows: 0-0.5 min, 30% B; 0.5-8 min, 30 to 50% B; 8-9 min, 50 to 95% B; 9-11 min, 95% B; 11-11.5 min, 95 to 30% B; 11.5-15 min, 30% B. Detection was carried out with a LCMS8060 mass spectrometer (Shimadzu) in the positive ionization mode. Of the four optimized MRM transitions per analyte, the most intense and specific Q1/Q3 pair was selected for quantification: 194 > 137 for acetic acid, 197 > 137 for D4-acetate, 208 > 137 for propionic acid, 210 > 137 for D2-propionate, 222 > 137 for butyric acid and 229 > 137 for D7-butyrate. The MRM parameters for all analytes and their internal standards are listed in Table S1. The concentration of each SCFA was determined by calculating its corresponding peak area ratio to that of the IS using a linear regression with 1/x weighting to the calibration curve.

### Histological analysis

Fixed proximal colon samples were paraffin-embedded, sectioned at 4 μm and stained with haematoxylin, eosin and saffron (HES). The slides were scanned using an automatic slide scanner (Nanozoomer, HAMAMATSU PHOTONICS K.K., Systems Division, Japan) and images obtained were analysed using NDP.view2 (version 2.8, HAMAMATSU PHOTONICS K.K., Systems Division, Japan) and ImageJ (version 1.53) [40] software. The lesions were quantified based on variations of tissue interface, which are obtained by the difference between the whole section and the luminal section.

### Statistical analysis

Statistical analysis of the data was performed using GraphPad Prism (version 5.00 for Windows, GraphPad Software, San Diego California USA, www.graphpad.com) and data are reported as arithmetic mean ± standard error of the mean (SEM). Statistical comparisons between experimental groups were performed using unpaired t-test or two-way ANOVA. A value of *p* < 0.05 was considered significant. When applied, the post-hoc test of Bonferroni was conducted if F value in ANOVA achieved statistical significance (*p <* 0.05) with no significant variance inhomogeneity. Partial least squares discriminant analysis (PLS-DA) score plots were made using the R Tidyverse package [41].

## Results

### MPA-induced enteropathy mouse model

**Fig. 1:**
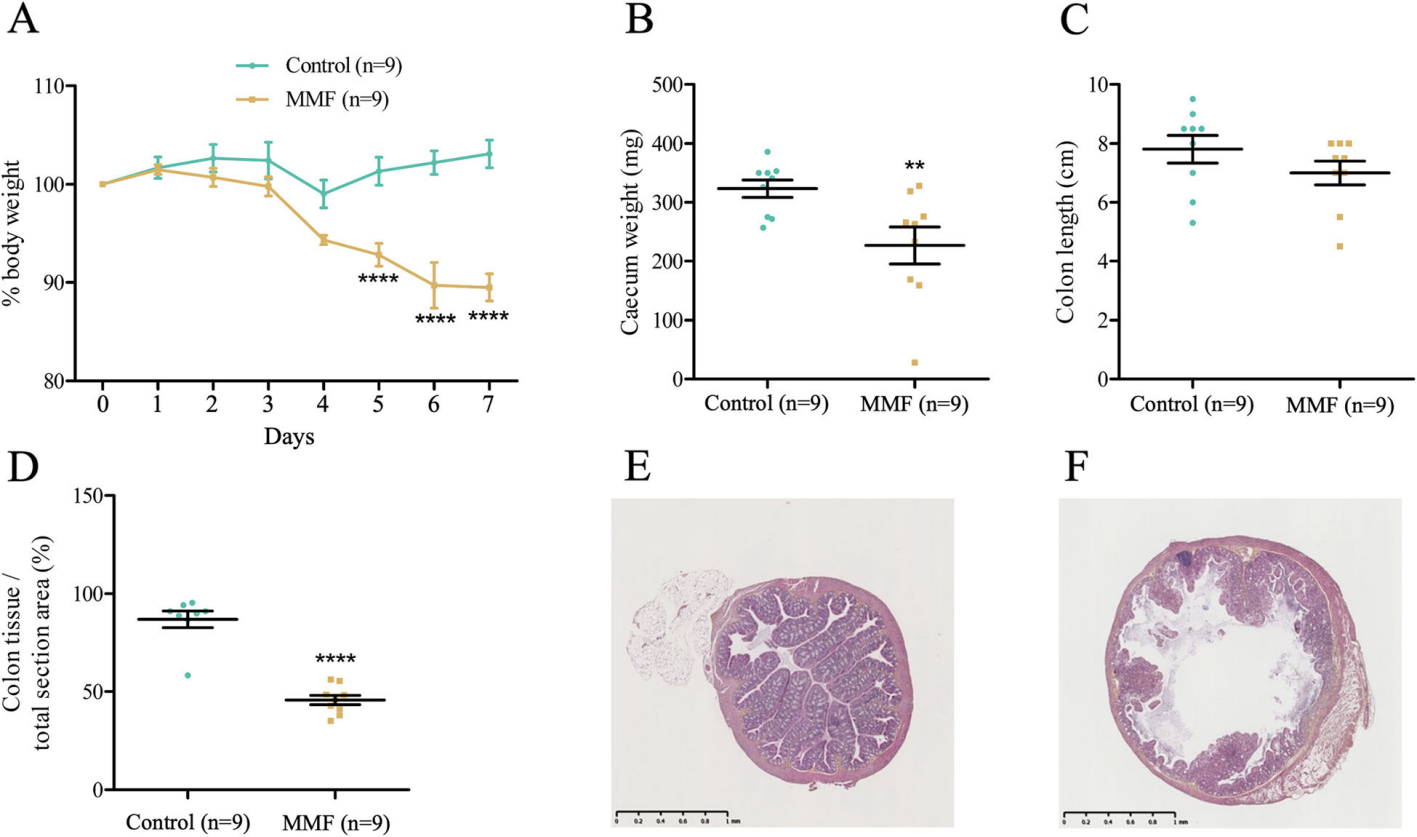
Mycophenolate-induced enteropathy model. MMF induces significant weight loss after five days of treatment in mice (**A**), a significant reduction in the caecum weight (**B**) and a slight decrease of the colon length (**C**). Histological analysis of proximal colon sections shows a significant decrease in the tissue area (**D**). Representative microphotographic cross-sections (HES staining) of the proximal colon of control (**E**) and MMF-treated mice (**F**). Scale bar equals to 1 mm. Two-way ANOVA followed by post-hoc Bonferroni multiple comparison test (**A**) and unpaired t-test analysis (**B**, **C**, **D**). **, *p* < 0.01; ****, *p* < 0.0001. *n =* number of mice

MMF treatment is associated with a significant weight loss starting at day 5 to reach approximately 10% at day 7 (control 103.1 ± 1.41% versus MMF 89.88 ± 1.28%, *p* < 0.0001) (Fig. 1A). This body weight loss is accompanied by a significant reduction in caecum weight (control 323.3 ± 14.87 mg versus MMF 226.8 ± 31.51 mg, *p* < 0.01) (Fig. 1B) and a slight, non-significant decrease in colon length (control 7.81 ± 0.47 cm versus MMF 7.00 ± 0.41 cm, *p* > 0.05) (Fig. 1C). Histological analysis revealed overall architectural disorganization and a significant decrease in the proximal colon tissue area (control 86.94 ± 4.21% versus MMF 45.68 ± 2.42%, *p <* 0.0001) (Fig. 1D). Microphotographic observations showed an almost complete destruction of the intestinal crypts and villi in MMF-treated mice in comparison to controls (Fig. 1E and F). Altogether, these data corroborate the development of a validated model of gastrointestinal toxicity in mice.

### SCFA profile alterations

#### Modifications in the digestive production of SCFA

**Fig. 2:**
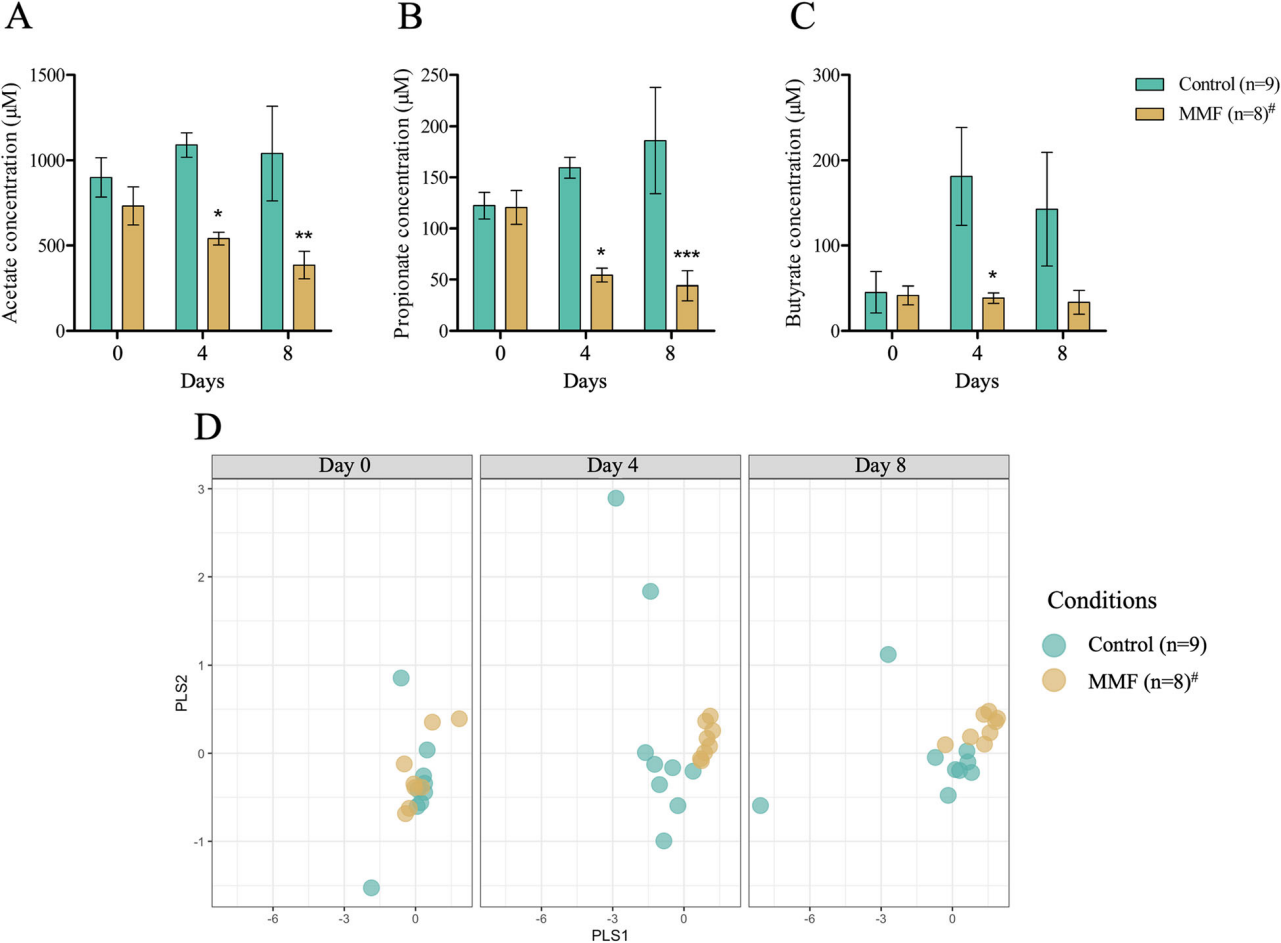
SCFA profile in faecal samples. Acetate (**A**), propionate (**B**), and butyrate (**C**) concentration in control and MMF-treated mice at days 0, 4 and 8. (**D**) PLS-DA score plot of all SCFA in control and MMF-treated mice according to time (days 0, 4 and 8). ^#^One missing data due to sample contamination. Statistical significance was assessed by two-way ANOVA followed by post-hoc Bonferroni multiple comparison test (**A**, **B**, **C**). *, *p* < 0.05; **, *p <* 0.01; ***, *p* < 0.001. *n =* number of mice

SCFA in faeces were quantified at days 0, 4 and 8 to investigate functional alterations of the gut microbiome induced by MMF. We found a significant decrease of acetate at day 4 (control 1090.5 ± 71.88 μM versus MMF 540.3 ± 37.6 μM, *p* < 0.05) and day 8 (control 1040.6 ± 278.161 μM versus MMF 384.7 ± 80.5 μM, *p* < 0.01) in MMF-treated mice in comparison to controls (Fig. 2A). The results was similar for propionate concentrations at day 4 (control 159.5 ± 10.27 μM versus MMF 54.34 ± 6.84 μM, *p <* 0.05) and day 8 (control 185.94 ± 51.96 μM versus MMF 44.07 ± 14.66 μM, *p* < 0.001) (Fig. 2B). Overall, there was no time-dependent modification in butyrate concentrations for MMF-treated mice despite fluctuation in the control group, leading to a significant difference only at day 4 (control 181.1 ± 57.4 μM versus MMF 38.55 ± 6.08 μM, *p <* 0.05) (Fig. 2C). The score plot of the PLS-DA model which includes concentrations of the three SCFA (acetate, propionate, butyrate) showed two groups at days 4 and 8 (Fig. 2D).

#### Modifications of SCFA plasma concentrations

**Fig. 3:**
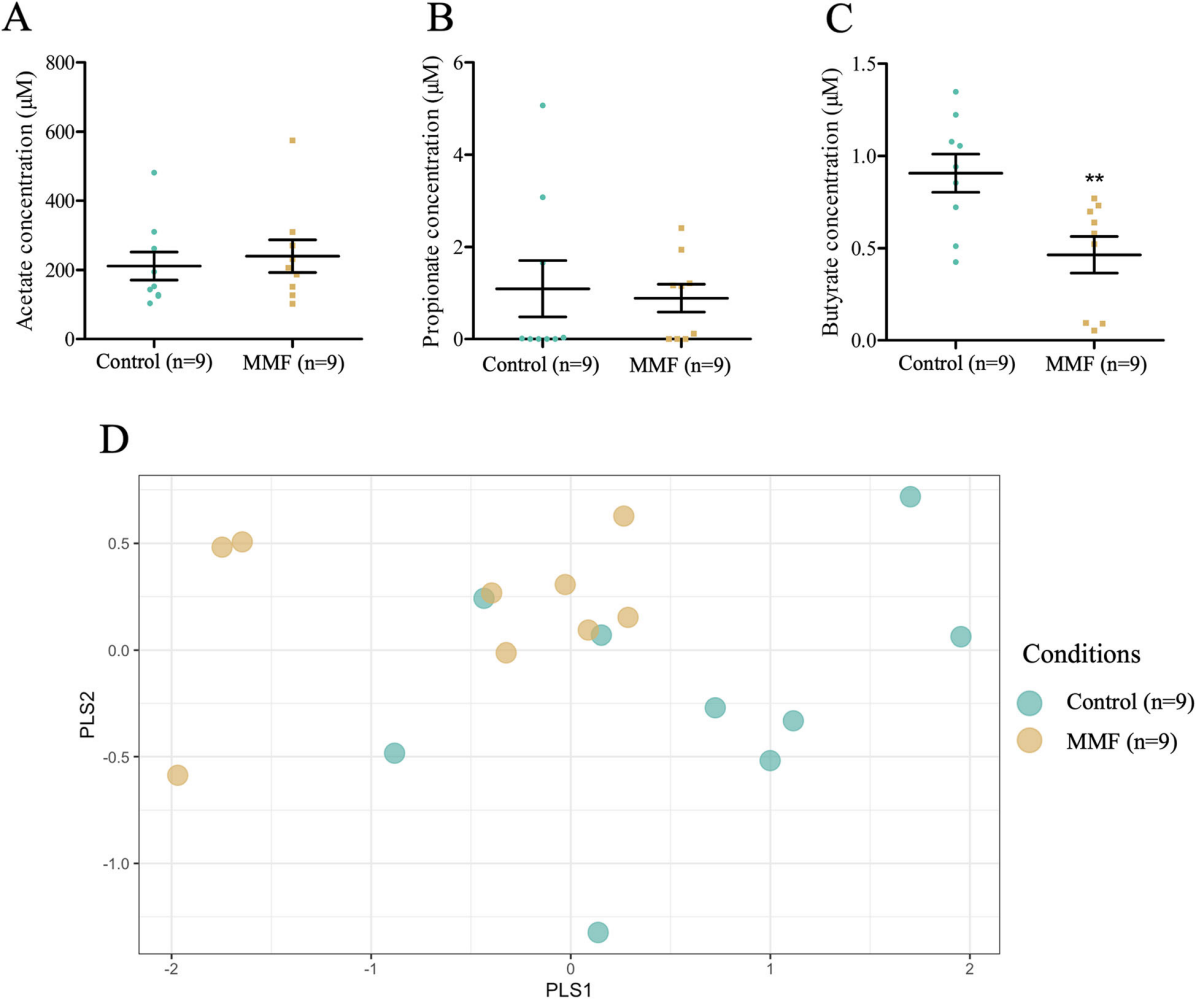
SCFA profile in plasma. Acetate (**A**), propionate (**B**), and butyrate (**C**) concentration in plasma of control and MMF-treated mice for 7 days. (**D**) PLS-DA score plot of all SCFA in control and MMF-treated mice. Unpaired t-test analysis (**A**, **B**, **C**). **, *p* < 0.01. *n =* number of mice

SCFA in plasma were quantified from samples obtained at day 8 after sacrifice. There was no change in acetate (control 211.5 ± 40.76 μM versus MMF 240.2 ± 47.41 μM, *p* > 0.05) or propionate (control 1.09 ± 0.61 μM versus MMF 0.89 ± 0.3 μM, p > 0.05) concentrations (Fig. 3A and B). In contrast, a significant decrease in butyrate concentrations was observed (control 0.91 ± 0.1 μM versus MMF 0.46 ± 0.1 μM, *p* < 0.01) (Fig. 3C). Again, the score plot of the PLS-DA model with the three SCFA showed two groups (Fig. 3D).

## Discussion

In the current study, we developed and validated a mouse model of mycophenolate-induced enteropathy that showed typical remodelling lesions of the colonic epithelium architecture. Detailed analyses of the concentration-time profile of the three main SCFA revealed for the first time a functional alteration of the gut microbiome associated with MPA.

### Severe enteropathy associated with MPA treatment

Our mice present an important decrease in body weight and exhibit typical and severe histological lesions, characterized by oedematous colonic mucosa with withering or marked crypt drop-out and villi destructions. These observations are consistent with the pattern of inflammatory injuries observed in treated patients with endoscopic evidence of ulcers and erosions [10–12]. Histologically, in these patients, the pattern of injury is typified by prominent crypt apoptosis with graft-versus-host-like-disease [8, 42, 43]. The caecum weight and colon length reduction could result from the direct impact of MPA on intestinal cells homeostasis [44].

### Alterations of the gut microbiome in mycophenolate-induced enteropathy

Recent reports have highlighted intestinal dysbiosis associated with MPA therapy [15, 16]. Intestinal dysbiosis has been linked to the decrease of the main pro-resolving mediators involved in the control of inflammatory processes and tissue damage [45, 46]. Our investigations thereby focus on the SCFA concentration-time profile and demonstrate digestive and systemic alterations in mycophenolate-induced enteropathy in mice. Indeed, there is a drastic reduction in digestive SCFA production of acetate and propionate that could contribute to the pathophysiology of mycophenolate-induced enteropathy. Along with this, it has been shown that germ-free or large spectrum antibiotic-treated mice show reduced proliferative activity of intestinal epithelial cells, which is reversed by SCFA supplementation [47]. Moreover, acetate, propionate and butyrate promote the development of intestinal organoids in vitro [47, 48]. In addition, increased gut microbiota SCFA production had a protective effect on a mouse model of acute experimental colitis [49, 50]. SCFA also diminished the development of tumours and attenuated colonic inflammation in a mouse model of colitis-associated colorectal cancer [51]. SCFA exert important roles in gut homeostasis and might be proposed as supportive therapy for patients treated with MPA.

Our data also demonstrate an important reduction of systemic plasma butyrate concentrations that might initiate pathological pathways in patients. Indeed, a close link between butyrate decrease and the onset of some cardiovascular or metabolic events was described [52]. For instance, de novo diabetes after solid organ transplantation is a complication that increases the risk of infections and graft failure in liver or kidney transplant patients [53, 54]. The immunosuppressive regimen also plays a critical role in the development of de novo diabetes, since tacrolimus and MMF strongly increase the incidence of new-onset diabetes after liver transplantation [55]. Butyrate supplementation promotes the mitigation of insulin resistance in animal models and protects against metabolic disorders [56, 57]. Regarding cardiovascular co-morbidities, arterial hypertension is frequently observed in kidney transplant patients [58]. Again, butyrate might exert a protective role since it suppressed angiotensin II-induced hypertension in a rat model [59].

Further investigations are needed to study the link between specific chronic diseases that require transplantation and the worsening of SCFA profile in post-transplantation periods. Moreover, the alterations of the gut microbiome induced by MPA could rationalize the progressive growth in the incidence of multidrug-resistance and extensively-drug-resistant strains in transplant patients [60, 61].

This will be helpful to decipher the specific impact of immunosuppressants. SCFA profile could be used as either as a predictive or a diagnostic biomarker for the follow-up of transplanted patients. SCFA supplementation might also be beneficial for treating mycophenolate-induced enteropathy.

## Conclusions

Our investigations reveal for the first time alterations of the SCFA time-profile in a mouse model of mycophenolate-induced enteropathy. This alteration could locally contribute to the inflammatory processes of mycophenolate-induced enteropathy. Moreover, decreased butyrate plasma concentrations might trigger pathological pathways leading to cardiovascular or metabolic comorbidities such as arterial hypertension or de novo diabetes in MPA-treated patients. Further investigations are needed to establish the molecular mechanisms and their relevance in human pathology. If these hypotheses are confirmed, SCFA can be envisaged in supplementation to improve the patients’ quality of life or as biomarkers of mycophenolate-induced enteropathy.

## Supplementary Information

**Additional file 1.**

## Data Availability

The datasets used and/or analysed during this study are available from the corresponding author on reasonable request.

## Abbreviations

(S)MCT: (Sodium-coupled) monocarboxylate transporter
3-NPH: 3-nitrophenylhydrazine
EDC: N-(3-dimethylaminopropyl)-N′-ethylcarbodiimide
FFAR: Free fatty acid receptor
GPCR: G-protein coupled receptors
HDAC: Histone deacetylase
HES: Haematoxylin, eosin and saffron
HPRT: Hypoxanthine-guanine phosphoribosyltransferase
IMPDH2: Inosine-5′-monophosphate dehydrogenase 2
MMF: Mycophenolic mofetil
MPA: Mycophenolic acid
PLS-DA: Partial least squares discriminant analysis
SCFA: Short-chain fatty acid
