# Supplementary material for: Alteration of the gut microbiome in mycophenolate-induced enteropathy: impacts on the profile of short-chain fatty acids in a mouse model
Source: BMC Pharmacol Toxicol. 2021 Oct 28;22:66. doi: 10.1186/s40360-021-00536-4 (PMC8555345; doi:10.1186/s40360-021-00536-4)
Supplement: Supplementary file 1 — Additional file 1. [file 40360_2021_536_MOESM1_ESM.docx]

**Supplementary material**

**Table S1.** Tandem MS parameters for short-chain fatty acids and internal standards. Quantification transition is indicated in bold.

| **Compound parameters** | | | | | | |
| --- | --- | --- | --- | --- | --- | --- |
| **SCFA-3-NPH derivatives** | **Precursor ion (m/z)** | **Product ion (m/z)** | **Dwell time (ms)** | **Q1 prebias (V)** | **Collision energy (V)** | **Q3 prebias (V)** |
| Acetic acid | **194.4** | **137.05** | **25** | **23** | **22** | **26** |
|  | 194.4 | 152.1 | 25 | 22 | 16 | 29 |
|  | 194.4 | 107.0 | 25 | 20 | 26 | 10 |
|  | 194.4 | 46.0 | 25 | 10 | 18 | 10 |
| D4-acetate | **197.5** | **137.1** | **25** | **24** | **20** | **29** |
|  | 197.5 | 153.1 | 25 | 10 | 17 | 14 |
|  | 197.5 | 107.1 | 25 | 14 | 24 | 13 |
|  | 197.5 | 150.05 | 25 | 10 | 16 | 13 |
| Propionic acid | **208.4** | **137.05** | **25** | **24** | **22** | **30** |
|  | 208.4 | 165.15 | 25 | 24 | 15 | 30 |
|  | 208.4 | 152.1 | 25 | 10 | 17 | 14 |
|  | 208.4 | 46 | 25 | 10 | 34 | 10 |
| D2-propionate | **210.3** | **137.15** | **25** | **24** | **22** | **28** |
|  | 210.3 | 107 | 25 | 23 | 26 | 29 |
|  | 210.3 | 167.15 | 25 | 24 | 15 | 25 |
|  | 210.3 | 153.1 | 25 | 23 | 15 | 14 |
| Butyric acid | **222.3** | **137.05** | **25** | **25** | **22** | **28** |
|  | 222.3 | 152.15 | 25 | 27 | 17 | 30 |
|  | 222.3 | 107.1 | 25 | 24 | 26 | 16 |
|  | 222.3 | 179.25 | 25 | 24 | 15 | 11 |
| D7-butyrate | **229.3** | **137.05** | **25** | **26** | **22** | **29** |
|  | 229.3 | 107.05 | 25 | 26 | 27 | 22 |
|  | 229.3 | 153.15 | 25 | 25 | 18 | 27 |
|  | 229.3 | 186.2 | 25 | 12 | 15 | 16 |
| **Source parameters** | | | | | | |
| **Nebulizer gas flow rate**  **(L/min)** | **Drying gas flow rate**  **(L/min)** | **Heating gas**  **(L/min)** | **Interface temperature (°C)** | **Heat Block temperature (°C)** | **DL temperature** **(°C)** | **CID gas pressure**  **(kPa)** |
| 3 | 10 | 10 | 300 | 400 | 250 | 350 |
